# Drug-likeness scoring based on unsupervised learning†

**DOI:** 10.1039/d1sc05248a

**Published:** 2021-12-14

**Authors:** Kyunghoon Lee, Jinho Jang, Seonghwan Seo, Jaechang Lim, Woo Youn Kim

**Affiliations:** Department of Chemistry, KAIST 291 Daehak-ro, Yuseong-gu Daejeon 34 141 Republic of Korea wooyoun@kaist.ac.kr; HITS Incorporation 124 Teheran-ro, Gangnam-gu Seoul 06 234 Republic of Korea jaechang@hits.ai; KI for Artificial Intelligence, KAIST 291 Daehak-ro, Yuseong-gu Daejeon 34 141 Republic of Korea

## Abstract

Drug-likeness prediction is important for the virtual screening of drug candidates. It is challenging because the drug-likeness is presumably associated with the whole set of necessary properties to pass through clinical trials, and thus no definite data for regression is available. Recently, binary classification models based on graph neural networks have been proposed but with strong dependency of their performances on the choice of the negative set for training. Here we propose a novel unsupervised learning model that requires only known drugs for training. We adopted a language model based on a recurrent neural network for unsupervised learning. It showed relatively consistent performance across different datasets, unlike such classification models. In addition, the unsupervised learning model provides drug-likeness scores that well separate distributions with increasing mean values in the order of datasets composed of molecules at a later step in a drug development process, whereas the classification model predicted a polarized distribution with two extreme values for all datasets presumably due to the overconfident prediction for unseen data. Thus, this new concept offers a pragmatic tool for drug-likeness scoring and further can be applied to other biochemical applications.

## Abstract 2

A new quantification method of drug-likeness based on unsupervised learning. The method only uses drug molecules as training set without any non-drug-like molecules.

## Graphical abstract

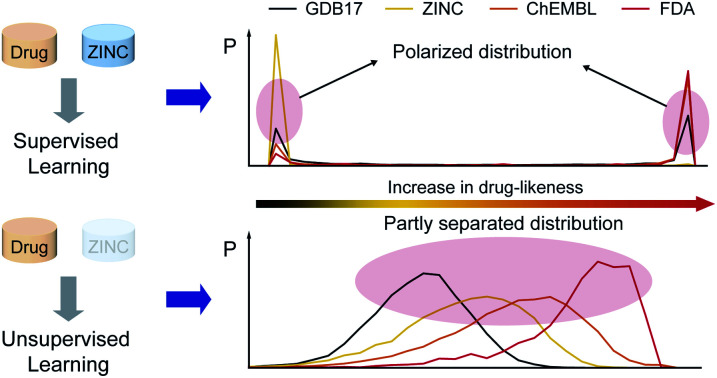

## 1 Introduction

Prediction of the biochemical properties of molecules is essential for efficient drug development. Simulations based on physicochemical principles can be used for this purpose. However, these methods are often not practical, especially if either target properties are associated with a number of different biological causes or those mechanisms are unclear. Data-driven approaches can be applied to such cases thanks to their convenience of making predictions from data alone without efforts to know the underlying biological mechanisms, as have been shown by successful examples of deep learning methods for accelerated drug developments.^1–7^

One example of such biochemical properties is drug-likeness. It can be used to remove compounds in advance that are likely to fail in clinical trials, which is important to enhance the success rate and reduce the economic costs of drug development.^8,9^ It is presumably associated with the whole set of essential characteristics to pass through clinical trials such as bioactivity, metabolic stability, toxicity, and so on. As those numerous factors can affect drug-likeness, it cannot be directly measured as a single-valued quantity. Therefore, various drug-likeness expressions have been suggested using data-driven approaches as a result of studies for more than two decades.^10^

In the beginning, the drug-likeness has been defined based on the certain physicochemical properties of the known drug molecules.^11^ In general, human experts select those physicochemical properties that seem to be closely associated with the drug-likeness and have been analyzed their distribution from drug databases. According to the result, a certain drug-likeness method is determined and used in a virtual screening scenario. Most methods developed in the early days were a classifier type based on the rules derived from the property distribution analysis.^12–20^ It is designed to determine whether a query molecule has drug potential or not. The representative example is the rules of five (Ro5) proposed by Lipinski *et al.*, which introduced the criteria of the number of hydrogen bond donors, the number of hydrogen bond acceptors, the molecular weight, and the octanol–water partition coefficient for being drug-like.^21,22^ Though these rule-based filters have been widely used thanks to the convenience, their inflexibility provokes a substantial possibility of screening out good drug candidates. For instance, 16% of the oral drugs violate at least one of the Ro5, and 6% of them violate more than two.^23,24^ Thus, these rules have been used to predict the bioavailability of molecules.

To prevent such strict cut-off, quantitative approaches have been proposed.^24–28^ The quantitative estimate of drug-likeness (QED) score is a representative example, which derives a final score from desirability functions fitted to the distribution of eight molecular properties.^23^ However, a recent study reported that QED is not practical to discriminate drug from non-drug molecules by showing that the distributions of the features used in QED are indistinguishable for drugs and non-drugs.^29^ This result indicates that human-driven features at the current level may have limitations for a screening purpose in practice.

To overcome the weakness of the previous data-driven methods, deep learning approaches have been attempted.^30,31^ The key difference of the deep learning approaches from the previous ones is to extract suitable features directly from raw data.^3,32^ Thus, the performance of deep learning models strongly relies on the quality and quantity of data used in training. Unfortunately, however, no quantitative data for the development of regression models is available because the drug-likeness is not a directly measurable quantity. In this regard, most deep learning approaches for drug-likeness adopted a two-class classification (TCC) method, which aims to classify query molecules into drug or non-drug.^29,33,34^

The TCC methods inevitably need both drug molecules for a positive set and non-drug molecules for a negative set. The positive set can readily be prepared with the known drug molecules. However, preparing a comprehensive negative set with chemical diversity is not straightforward, as true non-drug molecules can only be verified with the investigation through clinical trials. Thus, TCC approaches proposed so far regarded a certain set of molecules, so-called non-drug-like molecules, as the negative set by assuming that they have a very low probability of turning out to be a drug. Based on this assumption, Hu *et al.* used an autoencoder-based classifier to distinguish drug and ZINC molecules with ∼700 chemical descriptors that can be obtained from given molecules.^33^ Beker *et al.* improved the performance of the classifier to distinguish drug and ZINC molecules by combining autoencoder, Mol2vec, and graph convolutional network (GCN) models with uncertainty quantified from Bayesian deep neural network.^29^

In the TCC approaches, deep learning models are trained to find features from data that are appropriate for discriminating drugs from non-drug-like molecules. In other words, these TCC models tend to learn features specialized for discrimination between two classes rather than learn the respective features of drugs and non-drug-like molecules, which is in contrast to the traditional approaches that focused on deriving the common features of drugs. This implies that the features for discriminating drug and non-drug-like molecules learned by the TCC models can be significantly affected by the negative set for training. More seriously, TCC models trained with a specific negative set may not distinguish non-drug-like molecules that are substantially different from those in the negative training set. Indeed, Beker *et al.* pointed out that their TCC model predicted hydrocarbon molecules such as benzene and cyclohexane as drugs, even though they are obviously non-drug-like molecules.^29^ They argued that such a failure comes from the fact that the training data, which was a set of ZINC molecules, contains only a small portion of those hydrocarbons. It shows that TCC models may have limitations in generalization ability unless both the positive and negative sets encompass the entire chemical space of drug and non-drug-like molecules, respectively. In practice, it is not possible to prepare such an ideal data set, especially the negative one. In this regard, it is questionable whether or not the TCC models can be used in a practical virtual screening process.

Here, we propose a novel approach based on unsupervised learning to define drug-likeness in a data-driven way. Unlike supervised learning, such as the TCC model, unsupervised learning does not need any labeled data. It extracts features directly from unlabeled data, in our case, the known drug molecules. The unsupervised learning allows our model to find common features from the drug molecules. The trained model can be used to assess the drug-likeness of query molecules by analyzing how similar these molecules are to the known drugs in the resulting feature space. Because the proposed method only uses drug molecules in training, we expect it can avoid the aforementioned undesirable dependency on the negative sets of the TCC models originating from the use of an incomplete negative set. Conceptually, the unsupervised learning approach is rather close to the conventional approaches analyzing the common features of known drugs in the sense that it learns the feature space only from the drug molecules. However, it differs from the conventional ones in that it replaces hand-crafted features with those obtained by deep learning models.

Amongst others, we employed a simple language model based on recurrent neural network (RNN) for learning the probability distribution of the known drugs in an unsupervised way. The language model learns the probability of the next word from the previous sequence.^35^ After that, the trained model can generate new sequences or calculate the likelihood of the given sequence based on the Bayes' rule and the learned probability distribution. In the same way, the language model can be applied to the quantification of drug-likeness by representing drug molecules as a string such as SMILES. The model is trained to learn the probability of the next character from the given piece of SMILES. Then, the model can score the drug-likeness of query molecules in terms of the probability that those molecules will appear in the learned drug space.

To show the feasibility of our unsupervised learning for drug-likeness scoring, we have assessed its performance for several tasks. For comparison, we devised our own TCC model based on a graph convolutional network (GCN), which has been widely used in molecular property predictions,^36,37^ including drug-likeness, and examined its performance for the same tasks. We prepared various types of data sets, especially several negative sets to evaluate the generalization ability of the models. As expected, the language model showed relatively more consistent performance than the TCC model across different negative sets.

In what follows, we first describe the datasets used in this study. Then, we introduce how the language model can be used for drug-likeness scoring as a new concept and explain the TCC model for comparison with the implementation detail for both models. Finally, we analyzed the performance of the two models with tests on various datasets and drew conclusions.

## 2 Method

### 2.1 Dataset

Molecules in the datasets are represented with SMILES. For data preprocessing, we only used the molecules whose SMILES are RDKit^38^-readable, shorter than 100 characters, and represent a single molecule (SMILES without ‘·’).

We considered the known drug molecules as a positive set. We prepared two groups of the known drugs: the FDA-approved drug set (referred to as ‘FDA’) and the other approved drugs (referred to as ‘Worlddrug’). The Worlddrug and FDA sets were used for training and testing, respectively. For fair evaluation, we excluded molecules from Worlddrug if they have the Tanimoto similarity^39^ of 0.8 or higher with any molecule in FDA.

Negative sets were prepared from various databases: GDB17,^40^ ZINC15,^41^ and ChEMBL.^42^ GDB17 is a set of molecules composed of less than 17 C, N, O, S, and halogen atoms, which are virtually generated through a graph enumeration method. ZINC15 is composed of commercially available compounds for virtual screening in drug discovery. ChEMBL is a set of molecules that are known to be bioactive. For ChEMBL, we used molecules with a pChEMBL value of 5.85 or higher to only consider highly bioactive molecules. We used only molecules having a molecular weight of 600 or less in the ChEMBL's “small molecule” category to meet the SMILES length condition mentioned above. Molecules can be approved as a drug only if they successfully pass through all steps in drug development. As a drug candidate passes through one more step, it becomes more probable to be approved. Therefore, it is reasonable to assume that molecules at a later step in the drug development would be more drug-like than molecules at an earlier step. In this sense, within the prepared negative sets, GDB17 can be considered as the most non-drug-like dataset. ZINC15 could be the next non-drug-like dataset as it is regarded as a chemically accessible set for hit discovery. Finally, ChEMBL would be the least non-drug-like dataset among them.

Our language model requires only drug molecules and so was trained with Worlddrug. For the TCC model, we used the same Worlddrug as the positive set, while ZINC15 was used as the negative set in training since Beker *et al.* have shown that ZINC15 can be effective as a negative set of TCC models for drug-likeness study. To avoid a data imbalance problem, we randomly sampled 2833 molecules from ZINC15 for training, which is the same number as that of Worlddrug. As for the test set, we used FDA as the positive set and 10 000 molecules randomly selected from each negative set. Since the size of the test sets used here is small compared to the entire size of the negative sets (*e.g.*, GDB17 contains more than 166 billion molecules), we randomly sampled five different negative sets as our test sets and investigated whether different samplings cause different results.

The purpose of introducing drug-likeness is to remove molecules with a high failure rate before carrying out expensive experiments. Thus, it would be a more practical test to evaluate our models' performance on real drug candidates rather than relatively easy ChEMBL, ZINC15, and GDB17 cases. Here, we used the investigation group of the DrugBank^43^ database, which contains molecules in the clinical I/II/III stages. 1792 molecules that met the aforementioned structural conditions were obtained from the database and were called the “investigation” set. In fact, some of them may have high probabilities to be approved as a drug, meaning that, on average, they may have more common features with the known drugs. Therefore, the Investigation set cannot be readily judged as negative, unlike ChEMBL, ZINC15, and GDB17. It is intriguing to study how our TCC and unsupervised learning models predict the drug-likeness of those drug candidates. Table 1 summarizes all the datasets used in this work.

**Table 1:** Dataset information used in this work

	Data name	Composition	Description
Training	Worlddrug	2833 Worlddrug molecules	Dataset for training the RNN-based language model. The model only requires drug molecules
	Worlddrug/ZINC15	2833 Worlddrug + 2833 ZINC15 molecules	Dataset for training the GCN-based TCC model. ZINC15 has been widely used as a negative set for training TCC models for drug-likeness
Test	FDA/GDB17	1489 FDA + 10 000 GDB17 molecules	GDB17 is a set of molecules composed of less than or equal to 17 C, N, O, S, and halogen atoms generated by a graph enumeration method. It is expected to be highly non-drug-like
	FDA/ZINC15	1489 FDA + 10 000 ZINC15 molecules	ZINC15 represents a chemically accessible space, expected to be more drug-potential than GDB17
	FDA/ChEMBL	1489 FDA + 10 000 ChEMBL molecules	ChEMBL represents a bioactive space. Molecules with a pChEMBL value of 5.85 or higher were used and thus expected to be more drug-potential than ZINC15
	Investigation	1792 molecules from DrugBank that are under investigation	Examples of real drug candidates in clinical trials

### 2.2 Language model

A language model predicts the probability of the next possible characters based on the previous piece of sequence.^35^ In this work, we represent molecules as a sequence of characters with SMILES prepared as described above. For example, suppose the model takes the sequence ‘c1ccc’ as input, which can be a part of the cyclobutadiene's SMILES ‘c1ccc1’ or the benzene's SMILES ‘c1ccccc1’. Then, the model predicts the probability of ‘c’ and ‘1’ as output to be the next character. The output probability of each character in that example depends on the distribution of molecules that the model used as a training set. For instance, the predicted probability of ‘c’ would be higher than that of ‘1’, if it is trained with typical molecular databases such as PubChem, because benzene is a more common ring structure than cyclobutadiene in those datasets. In such a way, the model learns the probability distribution of molecules in the training set and predicts the probability of a query molecule as an output according to how frequently the features of the molecule appear in the distribution of the feature space obtained from the training data.

More precisely, the language model outputs a conditional probability distribution, denoted as *p*(*s*_*t*+1_|*s*_0_,…,*s*_*t*_), since it predicts the probability of the next possible characters (*s*_*t*+1_) from the given previous (*t* + 1) characters, denoted as *s*_0_, *s*_1_,…,*s*_*t*_. Similar to other language models, for the given SMILES, denoted as *S* = (*s*_1_, *s*_2_,…,*s*_*N*_), we used a sequence that begins with SOS (start of sequence), followed by the characters of SMILES, and ends with EOS (end of sequence). In other words, the sequence is defined as (*s*_0_, *s*_1_,…,*s*_*N*+1_), where *s*_0_ = SOS, *s*_*N*+1_ = EOS, for the given SMILES *S* = (*s*_1_, *s*_2_,…,*s*_*N*_). The model sequentially predicts the conditional probability *p*(*s*_*t*+1_|*s*_0_,…,*s*_*t*_), starting from *s*_0_ = SOS with *t* ≥ 0, until it meets *s*_*t*+1_ = EOS. For every *t* + 1-th step, the model is trained to predict those conditional probabilities as 1, when *s*_*t*+1_ is equal to the (*t* + 1)-th character of SMILES of the drug molecules, otherwise 0.

### 2.3 Drug-likeness scoring with unsupervised learning

**Fig. 1:**
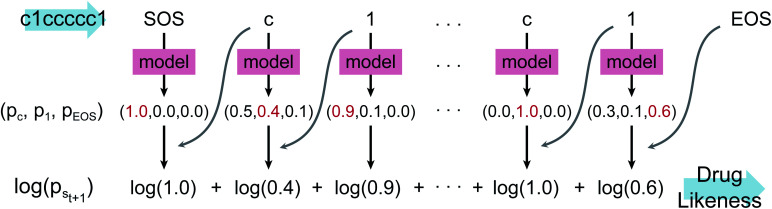
Diagram of calculating drug-likeness score from the language model. *s*_*t*_ denotes the *t*-th character of the given SMILES, *S*. *p*_*s*_*t*+1__ denotes the conditional probability distribution *p*(*s*_*t*+1_|*s*_0_,…,*s*_*t*_). By referring to the next character *s*_*t*+1_ (indicated as the grey curved arrows), the correct probability can be selected as shown in red. This example shows the scoring process of the drug-likeness of benzene. Starting from *s*_0_ = SOS, it repeats obtaining *p*_*s*_*t*+1__, until *s*_*t*+1_ equals to the EOS. The drug-likeness score is given as the sum of log values (log(*p*_*s*_*t*+1__)). Constant 100 is added to the score so that the maximum score becomes 100.

Fig. 1 depicts the overall scheme of drug-likeness scoring with unsupervised learning based on a language model. As the model learns the probability distribution, we can compute the joint probability, denoted as *p*(*s*_0_, *s*_1_,…,*s*_*N*_, *s*_*N*+1_), of a given sequence. Since this sequence is given as the SMILES of an input molecule, which is determined by the RDKit rule in this work, this joint probability can be defined as the final output probability of the given molecule. In short, *p*(*S*) = *p*(*s*_0_, *s*_1_,…,*s*_*N*_, *s*_*N*+1_). The joint probability can be calculated as the product of the conditional probability of each character consisting of the sequence according to the Bayes rule as follows.1
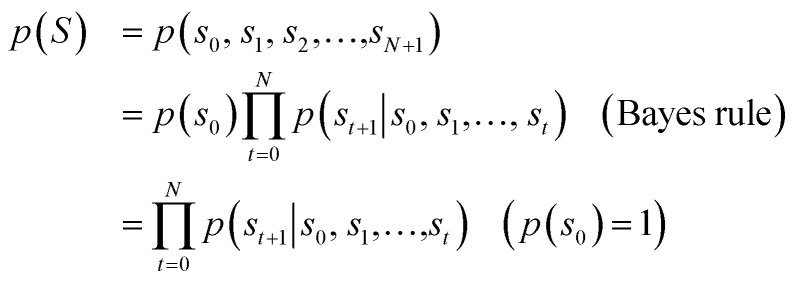
Here, *p*(*s*_0_) is equal to 1, because every sequence starts with SOS. Eqn (1) implies that the higher the value of each conditional probability, the higher the probability that the corresponding molecule will appear in the distribution of the training dataset. In terms of (structural or linguistic) syntax, the molecule has a high probability of having a common feature with molecules in the training set. In other words, *p*(*S*), defined as the product of conditional probabilities, means that the corresponding molecule to the SMILES code S as input is more likely to have the common features of the training set. Since we use drug molecules in Worlddrug as the training set for the language model, we can consider the *p*(*S*) value as a drug-likeness score. The actual *p*(*S*) value is numerically too small. For the sake of convenience, we applied the logarithm to the value and added 100 to the result, so the maximum score becomes 100. Our final drug-likeness score is defined as follows.2
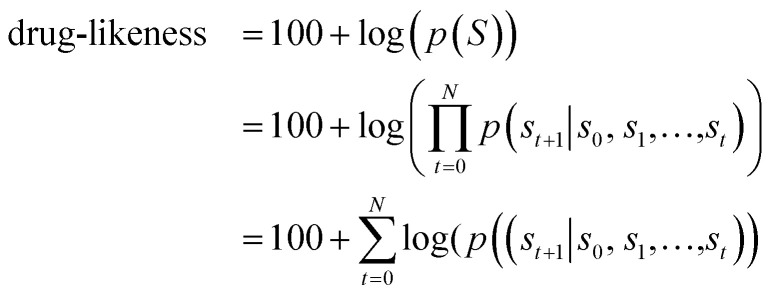


One of the important factors for drug-likeness is the information on stereocenters, as a slight change of the stereochemistry could lead to a huge difference in the drug-target interactions. Therefore, we considered all possible stereoisomers of given molecules for drug-likeness scoring and then chose the one with the highest score.

### 2.4 Implementation of the language model

**Fig. 2:**
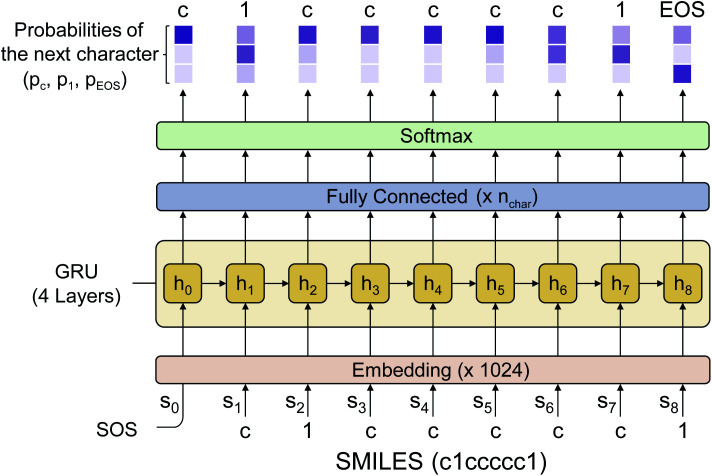
The architecture of the language model used in this work. It is a simple RNN consisting of four GRU layers and a fully connected layer which gives the final probability distribution of the possible next character throughout the softmax layer. The darker color in the output values means a higher probability. The numbers denoted in the embedding/fully-connected layer indicate the output dimension. The figure shows the working process of RNN with an input example of benzene represented as c1ccccc1, with *n*_char_ = 3.

Fig. 2 shows the language model used in this work. We adopted a simple RNN architecture based on the gated recurrent unit (GRU).^44^ The input starts with SOS, subsequently followed with the SMILES code of a given molecule, and ends with EOS. Considering all stereoisomers can cause very slow training, so we used only a single stereoisomer randomly sampled among them at each training epoch. Also, the drug-likeness based on the language model depends on the SMILES format since the probability of the next character is given conditionally by the previous characters. Therefore, we only used the canonical SMILES format to avoid it. The output of the GRU is passed through a fully connected layer and a softmax layer to convert the output value to the probability distribution of the next character. In this work, we used four GRU layers with the dimension of the hidden state equal to 1024. The output dimension was equal to 66 since there are 66 different types of characters (including EOS) within SMILES for the entire molecules of the PubChem database. The loss is calculated by the cross-entropy between the model's output and the encoded one-hot vectors of a given sequence. We also tested a transformer model,^45^ another widely used language model, but its performance was poorer than that of the RNN for this drug-likeness problem. Thus, we will only discuss the RNN-based language model and its results in what follows.

### 2.5 Transfer learning

Transfer learning is useful to improve the performance of a model, especially if the training data on a given task is insufficient.^46^ The model is first trained on a general task with a large amount of data and then fine-tuned on a target task that needs to be solved with a small amount of data. The model performance can be improved as the pre-trained model already learned relevant features from a large amount of data on the general task. Since the number of known drug molecules is not enough, we applied the transfer learning strategy to training the RNN-based language model. We performed pre-training it with 10 million molecules that were randomly selected from the PubChem database^47^ that covers a wide range of general molecules. After that, we fine-tuned the model parameters with the drug molecules in Worlddrug. We note that the transfer learning improved the model performance significantly; we refer to ESI† for more details about the effect of the transfer learning. Therefore, we only use the results from the model with transfer learning hereafter.

### 2.6 Graph convolution network

**Fig. 3:**
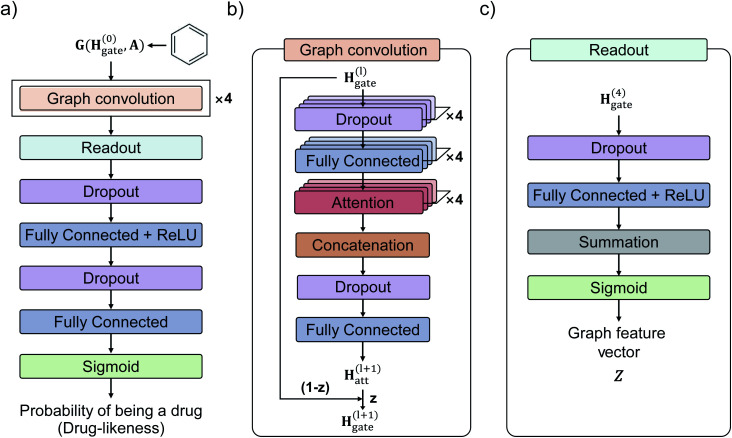
Illustration of the GCN model with attention and gate-augmentation mechanism for two-class classification. (a) The overall model architecture. The model consists of four graph convolution layers, a single readout layer, two fully-connected layers, and a sigmoid layer which gives a probability of being a drug as an output. (b) Each graph convolution layer uses the attention and gate-augmentation mechanism. (c) The readout layer aggregates the atom features as a graph feature vector.

Fig. 3 shows the GCN-based TCC model tested in this work. We used the attention and gate-augmented GCN that was proposed in our previous work.^48^Fig. 3(a) shows the overall scheme of the model. It receives the graph representation of molecules, denoted as **G**(**H**^(0)^, **A**), where **H**^(0)^ is the initial atom feature, and **A** is the adjacency matrix of a given molecule. The input passes through four graph convolution layers. Then, the resulted output of the graph convolution layers passes through the readout layers, which aggregate the set of vectors into a single graph feature vector representing the features of the whole graph. Finally, the graph feature vector passes through two MLP layers where the dimension of the first output is 256, and the second one is 1. The final output of the MLP layers is converted to a probability of being drug through a sigmoid function. Fig. 3(b) illustrates the structure of a single graph convolution layer, which uses the attention and gate-augmentation mechanism. Each convolution layer has four fully connected layers, four self-attention heads, and one fully connected layer. Fig. 3(c) shows the readout layer. The readout layer first sums up all the updated node features and then produces a graph feature vector with the dimension of 256. We put dropout layers right before the fully connected layers as a regularization for preventing overfitting.

To train the GCN-based TCC model, we used Worlddrug and ZINC15 as a positive set and a negative set, respectively. We used binary cross entropy as a loss function, so the model was trained to classify whether a query molecule is a drug or non-drug-like. For the input atom feature of each node, we included an atomic symbol, number of hydrogen atoms, degree, implicit valence (prepared by RDKit), and aromaticity as summarized in Table 2. The edge feature, *i.e.*, the bond connectivity, can be written as an adjacency matrix. We used a 5-fold cross-validation method for training since the size of the training set was small (<10 000). More details of the training hyperparameters of our models are available in ESI.†

**Table 2:** The atom features of the input for the GCN-based TCC model

Atom feature	Type	Values
Symbol atom-type)	One-hot	C, N, O, S, F, P, Cl, Br, I, else
Number of hydrogens	One-hot	0, 1, 2, 3, 4, 5, else
Degree (without hydrogen)	One-hot	0, 1, 2, 3, 4, 5, else
Implicit valence (RDKit)	One-hot	0, 1, 2, 3, 4, else
Aromaticity	Integer	0, 1

## 3 Results and discussions

### 3.1 Performance of our drug-likeness models on FDA/ZINC

We first examined the performance of the GCN-based TCC model and the RNN-based unsupervised learning model on a classification test using FDA/ZINC15, a typical test set used in other studies.^29,33^ QED was also included in the test for comparison. We evaluated the AUROC of each model for the same task.

**Fig. 4:**
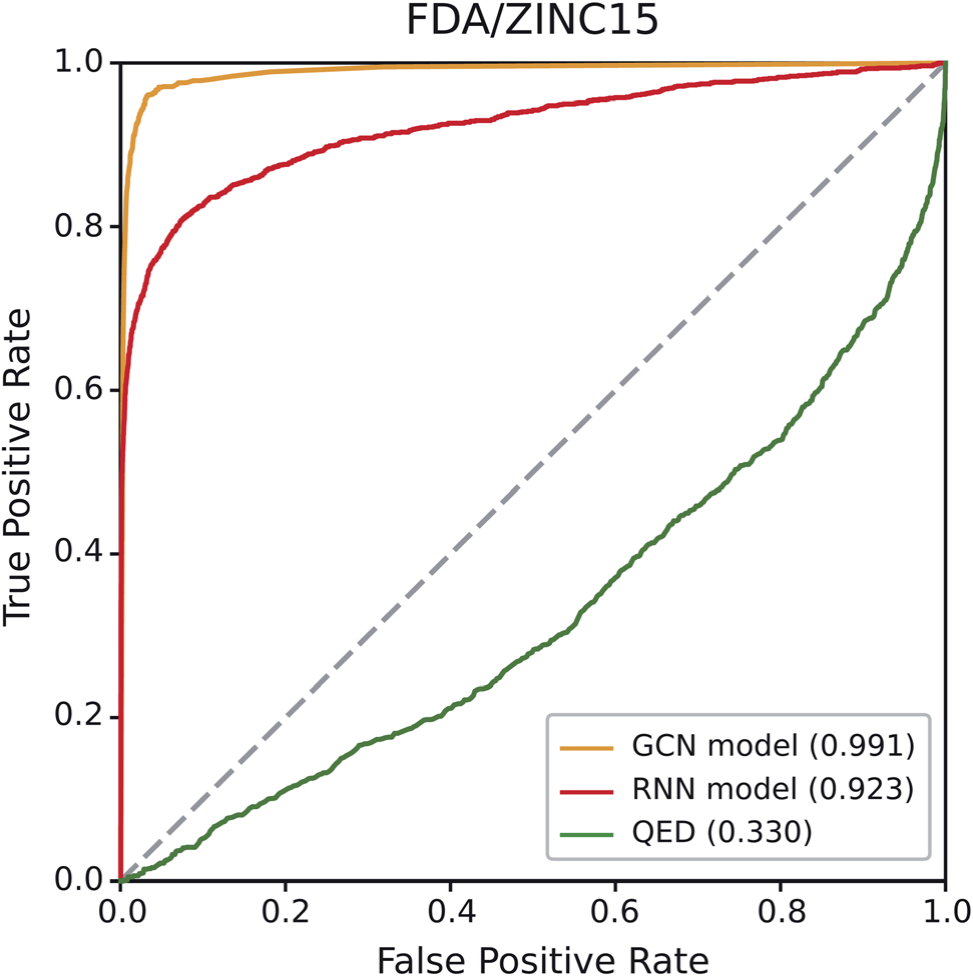
ROC curves of the three drug-likeness models on the FDA/ZINC15 test set. The values in the legend represent the AUROC value of each ROC curve.

Fig. 4 shows the resulting ROC curves, and the corresponding AUROC values are given in the legend. The grey dotted line indicates the ROC curve of a random classifier whose AUROC equals to 0.5. Notably, the two deep learning models showed high AUROC values above 0.9, whereas that of QED (0.330) was even worse than the random classification. These results indicate the high potential of deep learning compared to traditional data-driven approaches based on hand-crafted features. Of the two deep learning models, the GCN model performed slightly better than the RNN model. The latter performed well with a high AUROC value of 0.923, while the former showed an almost perfect AUROC value close to 1 (0.991). It is not surprising because the GCN model was trained with ZINC15 as a negative set. If the feature difference between positive and negative samples in a test set is similar to those in a training set, it can readily be identified by the TCC model, leading to high prediction accuracy. On the contrary, the RNN model, which was trained in the way of unsupervised learning, has never seen any molecule in ZINC15 during the training; it has been trained only with Worlddrug as a true drug set. The model does not explicitly learn the difference between positive and negative samples. Instead, it evaluates to what extent the features of molecules in either FDA or ZINC15 share with those of Worlddrug. Thus, the high AUROC of the RNN model means that the molecules of FDA had much more common features with those of Worlddrug compared to those of ZINC15. However, the AUROC of the RNN model (0.923) also implies that ZINC15 molecules also contain a slight extent of the common features of Worlddrug molecules, leading to a slightly lower performance than the GCN model.

### 3.2 Data dependency of the model performance

Next, we studied how consistent the performances of the three models are across different datasets. This test is important for their practical use in virtual screening, especially considering the fact that such models are trained with a tiny portion of the entire chemical space of potential drug candidates. To assess the generalization ability of our models, we evaluated the model performance by changing the negative set to GDB17, ZINC15, and ChEMBL, while using the same FDA positive set.

Table 3 shows the mean AUROC values along with the standard deviation of each model during the five tests on GDB17, ZINC15, and ChEMBL negative sets. The standard deviation value is relatively small compared to each mean value, meaning that there is no significant change in the performance of the model. Therefore, we used results from a single test for each data type throughout the article except for Table 3. The bold values denote the highest AUROC value for each test set. Except for FDA/ZINC15 discussed before, the RNN-based unsupervised learning model outperformed the GCN model and QED. The RNN model showed relatively high values with the gradual decrease from GDB17 to ZINC15 to ChEMBL, whereas QED underperformed consistently. In contrast, the performance of the TCC model was significantly decreased in FDA/GDB17 and FDA/ChEMBL compared to FDA/ZINC15. It should be noted that the AUROC value of the GCN model for FDA/GDB17 was even lower than FDA/ZINC15, although molecules in GDB17 can be regarded as more non-drug-like. This result meets our expectation that the features learned in the TCC model are particularly over-fitted for distinguishing FDA and ZINC15, but they may not work well with other test sets. In that sense, unsupervised learning would be a more pragmatic approach for drug-likeness by avoiding the data dependency problem of the TCC model. In addition, we investigated whether the data dependency is common to other data types of negative training sets. We trained each model with a negative set sampled from various data types and carried out the same test with that of Table 3. The result (available in ESI†) confirmed that the TCC model shows a similar data dependency to that of Table 3 regardless of the data type.

**Table 3:** AUROC values of the three drug-likeness models on different test sets with respect to the negative sets. The maximum value for each test set was written in bold

Model	FDA/GDB17	FDA/ZINC15	FDA/ChEMBL
RNN (unsupervised)	**0.979 ±** **0.005**	0.921 ± 0.001	**0.824 ± 0.010**
GCN (TCC)	0.747 ± 0.002	**0.991 ± 0.000**	0.701 ± 0.012
QED (traditional)	0.539 ± 0.024	0.326 ± 0.003	0.549 ± 0.004

The result in Table 3 may vary since we only used a small fraction of the negative training set sampled randomly. To examine such undesirable data dependency, we performed two additional experiments. First, we trained the model with five different negative sets that have been sampled independently from the same data type and assessed its performance. Second, we applied a PU learning approach by regarding the negative set as unlabeled since the fixed negative set we assumed may contain positive samples. The PU learning enables to label reliable negative samples out of the unlabeled data automatically, so it is expected to alleviate the data bias stemming from the fixed negative set. We adopted the PU learning method proposed by Fusilier *et al.*^49^ In both experiments, we found no significant change in the model performance from the original result in Table 3. For more details of the experiments, we refer to the ESI Section.†

### 3.3 Feasibility of drug-likeness scoring

Another expected advantage of unsupervised learning is that it can naturally quantify drug-likeness from the learned distribution of drug molecules. To test the feasibility of using the learned probability distribution of the model as drug-likeness scores, we analyzed the distribution of the output values of each model for molecules in each test set. The RNN model quantifies the drug-likeness score of a query molecule using eqn (2). On the other hand, the GCN model works as a classifier, so its output can be interpreted as the probability that a query molecule belongs to the positive class. Thus, we regarded the probability values as drug-likeness scores despite that there would be a debate on whether the output value of the TCC model can be used as an actual probability.^50–52^ QED was also used for comparison.

**Fig. 5:**
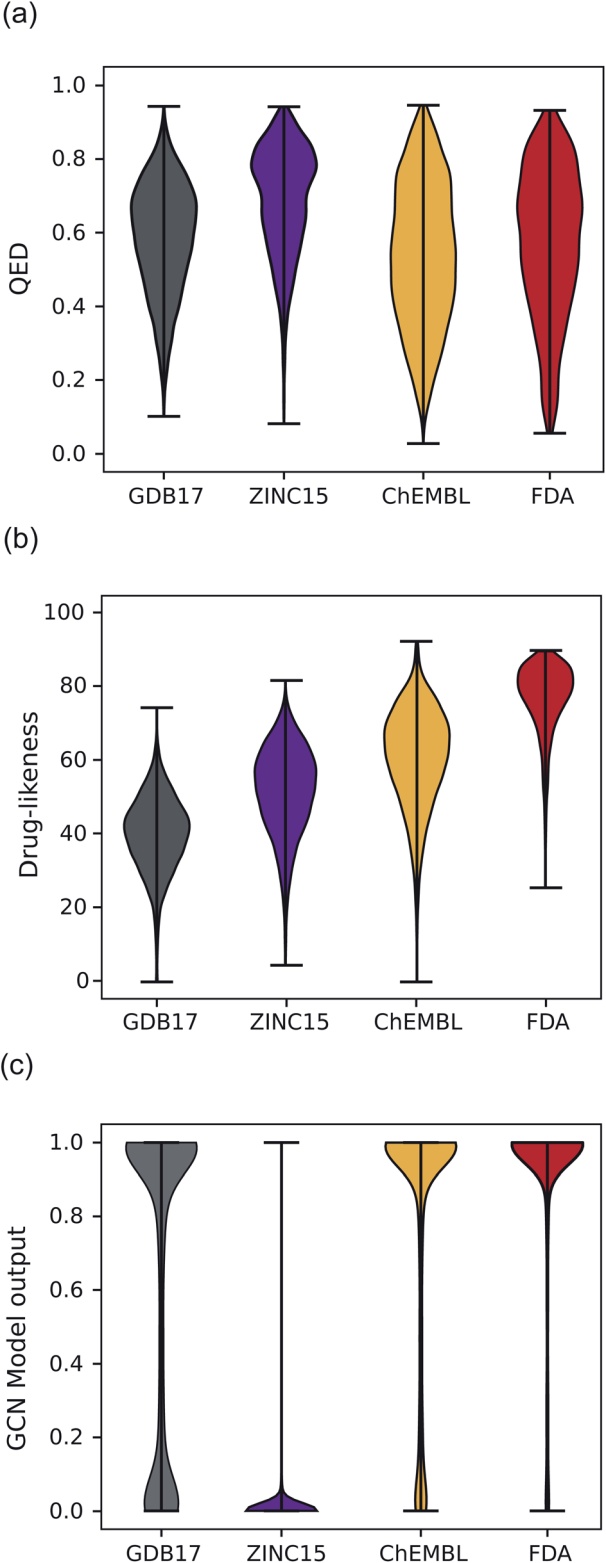
The violin plots of drug-likeness scores on various datasets. (a) The drug-likeness score distribution of QED. (b) The drug-likeness score distribution of the RNN-based unsupervised learning model. (c) The drug-likeness score distribution of the GCN-based TCC model.

Fig. 5 shows the prediction results of QED, the RNN model, and GCN-based TCC model. As a baseline, QED failed to discriminate the drug molecules in FDA from the other non-drug-like molecules, as shown in Fig. 5(a). It evaluated ZINC15 even as the most drug-like data set. One possible reason for the remarkably high score is that ZINC15 has been made to obey Ro5 for use in drug discovery. QED uses the features of Ro5 to evaluate drug-likeness score and hence it will score high if test molecules obey Ro5. The result once again confirmed the previous report that the features QED adopted were not effective for drug-likeness scoring.^29^

In contrast, the RNN model gave separated distributions for each case. Fig. 5(b) shows the results. The average output value of the RNN model for each dataset gradually increases from the lowest value of GDB17 to the highest value of FDA. The drug-likeness distribution form of GDB17 is close to a typical Gaussian distribution, presumably due to its nature as a collection of randomly generated molecules. On the contrary, the distribution form of the FDA is substantially distorted from a Gaussian function toward higher values, which well reflects the fact that the FDA molecules are true drugs. Interestingly, the distribution shapes of ZINC15 and ChEMBL are closer to a Gaussian function but with slight distortions toward higher values; that of ChEMBL shows more distortion than that of ZINC15. This propensity agrees with our expectation considering the characteristics of each dataset as discussed in the dataset section; GDB17 as the most non-drug-like, subsequently followed by ZINC15 and ChEMBL. This result also supports our suggestion that the drug-likeness defined as eqn (2) can be a reasonable alternative as a pragmatic tool to conventional ones.

Notably, the score distributions given by the TCC model were polarized to either 0 or 1 for all the datasets as shown in Fig. 5(c). It gives well-discriminated scores for ZINC15 and FDA. However, it failed to discriminate the other negative test sets from FDA. This result is another evidence that the TCC model was trained to discriminate ZINC15 and FDA only, so it cannot be generalized to other datasets. Moreover, the TCC model may cause overconfident judgments for unseen data. For example, it predicted high probabilities above 0.9 for 53% of the GDB17 molecules, whereas it gave very low values below 0.1 for most ZINC15 molecules. This is because the GCN model has used ZINC15 as the negative training set. It tends to make overconfident judgments for unseen molecules. In fact, such overconfident prediction by the TCC model has been reported in other domains as well.^53,54^

**Fig. 6:**
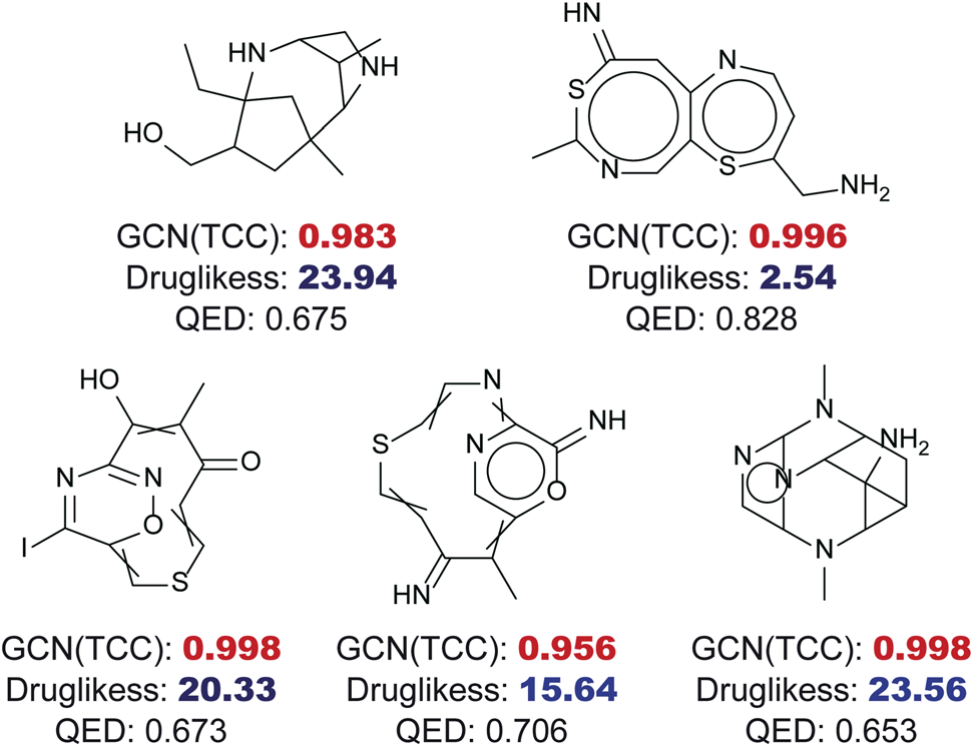
Examples of overconfident predictions of the GCN-based TCC model within the GDB17 dataset, a set of highly non-drug-like molecules. The GCN output probabilities of those molecules are written as bold with red color, where all of them are larger than 0.95. The drug-likeness scores obtained by the RNN-based language model are written as bold with blue color, where all of them are less than 25, fairly small compared to scores of drug molecules. QED also showed slight overconfidence, where all of the QED values were between 0.6 and 0.8.

Fig. 6 shows five randomly selected molecules from GDB17 that had extremely low drug-likeness scores, less than 25, predicted by the RNN model but had high probabilities above 0.95 estimated by the GCN model. While the RNN model scored them as non-drug-like, which seems reasonable, the GCN model strongly predicted them as drug-like as a result of overconfidence. QED also assigned relatively high values to those molecules ranging from 0.6 to 0.8.

### 3.4 Testing models on virtual screening scenario

As the final test, we applied our models to the investigation set consisting of molecules in clinical phases. This task is closer to a real virtual screening scenario that aims to select compounds from numerous candidates with considerable potential to pass through all clinical trials. Classifying them into drugs and non-drugs is challenging because they have relatively high probabilities of being approved drugs than the other negative sets presented in this work and thus may share more common features with the known drugs. We compared the drug-likeness score for the molecules in the investigation set with those of ChEMBL and FDA.

**Fig. 7:**
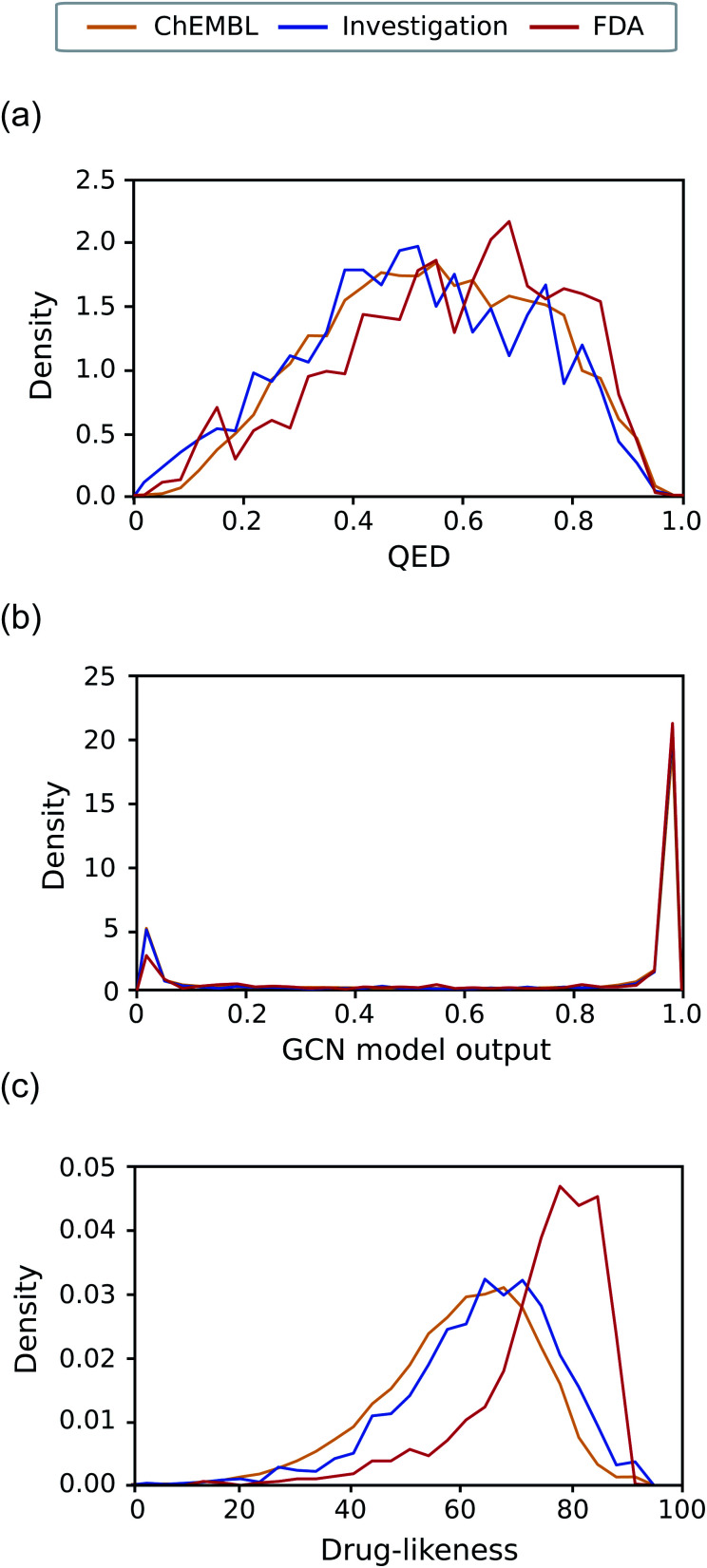
Distribution of the drug-likeness scores of the investigation set. The distribution of ChEMBL and FDA were also plotted for comparison. (a) The distribution of QED. (b) The distribution of the output of the GCN-based TCC model. (c) The distribution of the drug-likeness scores from the RNN-based language model.

Fig. 7(a)–(c) exhibits the distributions of the drug-likeness predicted by QED, the GCN-based TCC model, and the RNN-based unsupervised learning model for the three datasets, respectively. Both QED and GCN models provided indistinguishable distributions across the three datasets, manifesting their limitations for practical use. The QED scores show very similar distributions for all the datasets, covering a broad range from 0 to 1, already shown in Fig. 5(a). In contrast, the GCN model resulted in polarized distributions to either 0 or 1 for all the datasets, as observed in Fig. 5(c).

Meanwhile, the RNN-based unsupervised learning model made a meaningful difference in the distribution of the three test sets. The distribution form for the investigation set looks more similar to that of ChEMBL. We suspect that the relatively low discrimination between the ChEMBL and investigation sets may be due to limitations in unsupervised learning. It only evaluates how similar molecules in the test set are to those in the training data (in this case, Worlddrug). If the two test sets have almost equal extent of similarity to the training set, they may have similar drug-likeness scores, even though they are different from one another. Nonetheless, the investigation set has more portions at higher values, which follows the propensity observed in Fig. 5(b) Compared to the FDA case, however, the investigation set had more molecules in lower values (less than 60). On average, the RNN model placed the investigation set between ChEMBL and FDA. This prediction result can be rationalized considering the fact that only less than 10% of the drug candidates in clinical trials can eventually be approved as drugs,^5,31,55^ while the ChEMBL molecules have a much lower success rate. It should be noted that the RNN-based drug-likeness score proposed here has not been proved to be directly interpreted as the real probability of being approved. However, it has shown the feasibility as an alternative to conventional drug-likeness scoring methods.

## 4 Conclusion

Here we proposed a new approach based on unsupervised learning for drug-likeness scoring. A recurrent neural network (RNN)-based language model was adopted for implementation. We also implemented a graph convolution network (GCN)-based two-class classification (TCC) model for comparison. We tested the two deep learning models on various test sets, including GDB17, ZINC15, ChEMBL as a negative set and approved drug molecules in the FDA and Worlddrug databases as a positive set. We also considered the investigation group (investigation set) in the DrugBank database, which contains molecules in clinical phases. The GCN-based TCC model showed excellent performance when a test set was similar to a training set. However, its performance severely degraded for the other test sets. In contrast, the RNN-based unsupervised learning model showed relatively low data dependency. It determines the drug-likeness of a query molecule by identifying to what extent the molecule shares the common features of the drug molecules used in training. Thus, it can avoid such a strong dataset dependency observed in the TCC model. In addition, the unsupervised learning model well-separated drug-likeness distributions with increasing mean values in the order of GDB17, ZINC15, ChEMBL, Investigation set, and FDA. This result meets our expectation that molecules at a later step in a drug development process would be more drug-like than those at an earlier step. However, the TCC model predicted a polarized distribution with two extreme values (0 or 1) for all datasets, presumably due to the well-known overconfidence problem. Therefore, unsupervised learning can be a practical approach for drug-likeness scoring as an alternative to conventional methods.

In this study, we represented molecules with SMILES to implement the RNN-based unsupervised learning model. The drug-likeness score is given as the sum of the log values of each conditional probability output of the RNN model. It should be noted that the RNN model tends to give a higher drug-likeness score for molecules with shorter SMILES codes regardless of their structures, presumably due to numerical issues. For example, methane (obviously non-drug-like molecule), represented as ‘C(EOS)’ in SMILES, can have a high drug-likeness score. Similarly, molecules with longer SMILES codes can have lower drug-likeness scores since the conditional probability of each character in SMILES, which should be smaller than one, is multiplied many times. We expect such weakness can be resolved by using other unsupervised learning models with better model architectures and molecular representation.

Apart from that, the idea of using unsupervised learning for the drug-likeness prediction can be readily applied to other problems in which only one class of data is primarily available. Toxicity, metabolic stability, or synthetic accessibility can be such examples. We believe that our concept proposed here offers new opportunities to solve biochemical problems using a deep learning approach.

## Data availability

The source code and datasets used in this article can be found at https://github.com/SeonghwanSeo/DeepDL.

## Author contributions

Conceptualization: J. L. and W. Y. K.; methodology: J. L.; software, investigation and formal analysis: S. S., J. J. and K. L.; writing – original draft: S. S., J. J. and K. L.; writing – review & editing: K. L. and W. Y. K.; supervision: W. Y. K.

## Conflicts of interest

There are no conflicts to declare.

## Supplementary Material

SC-013-D1SC05248A-s001

SC-013-D1SC05248A-s002

SC-013-D1SC05248A-s003
