# Supplementary figures and images for: Drug-likeness scoring based on unsupervised learning
Source: Chem Sci. 2021 Dec 14;13(2):554–65. doi: 10.1039/d1sc05248a (PMC8729801; doi:10.1039/d1sc05248a)

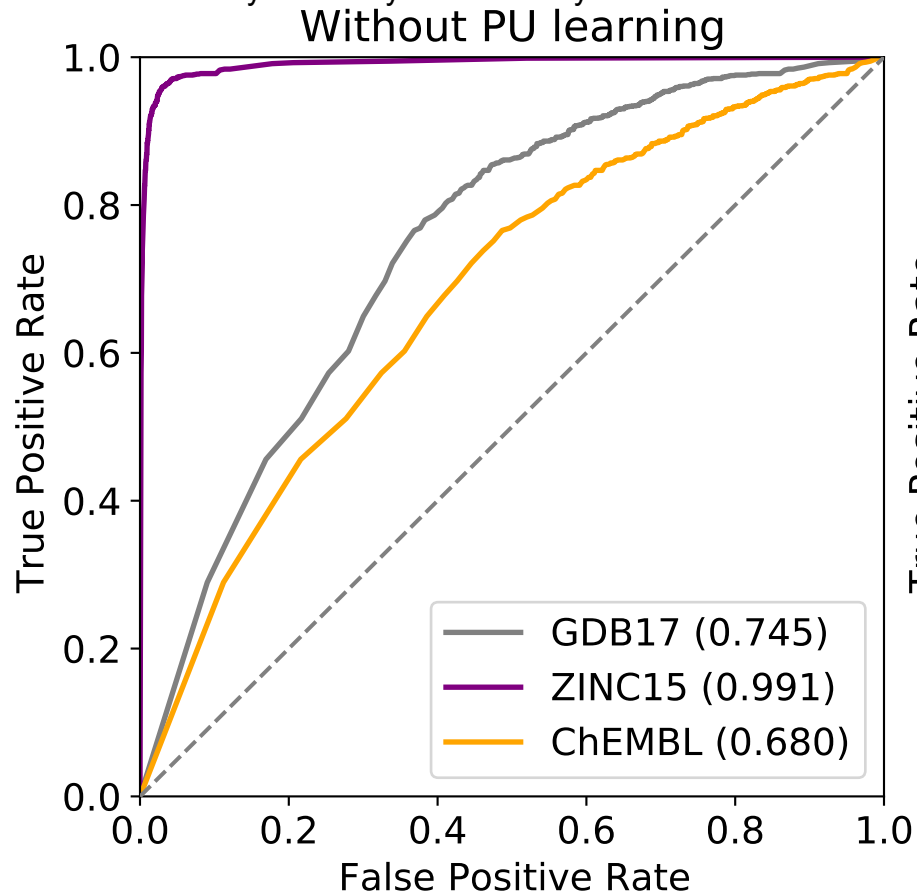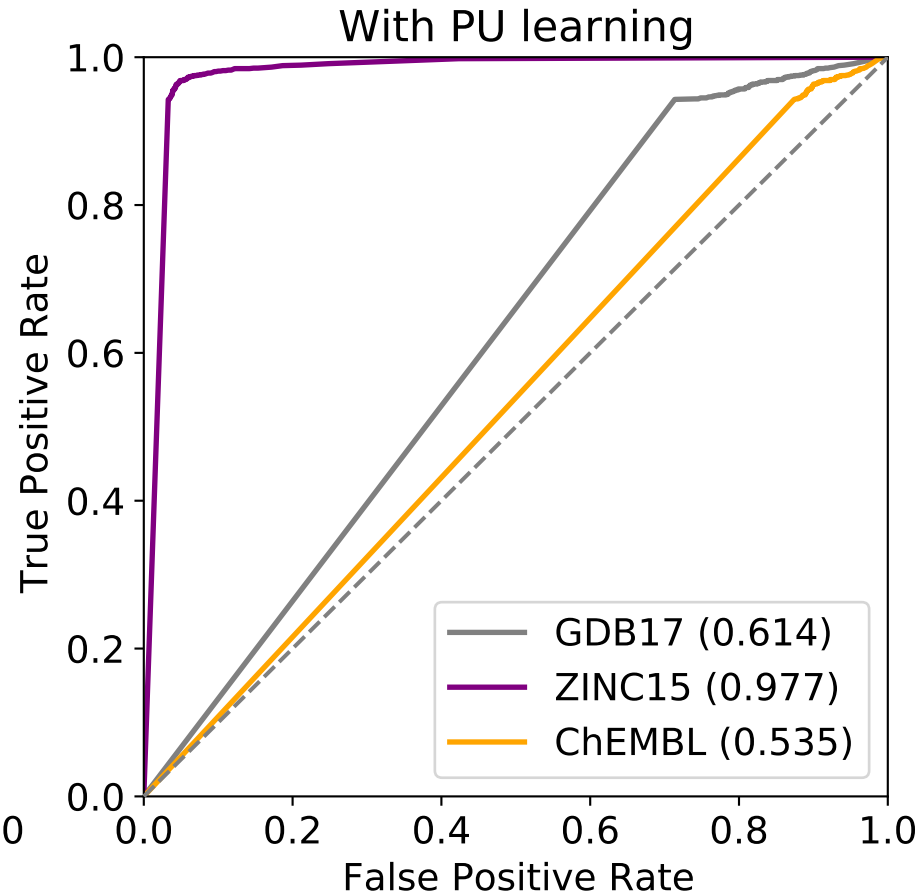

Supplement: SC-013-D1SC05248A-s001 [file SC-013-D1SC05248A-s001.pdf]

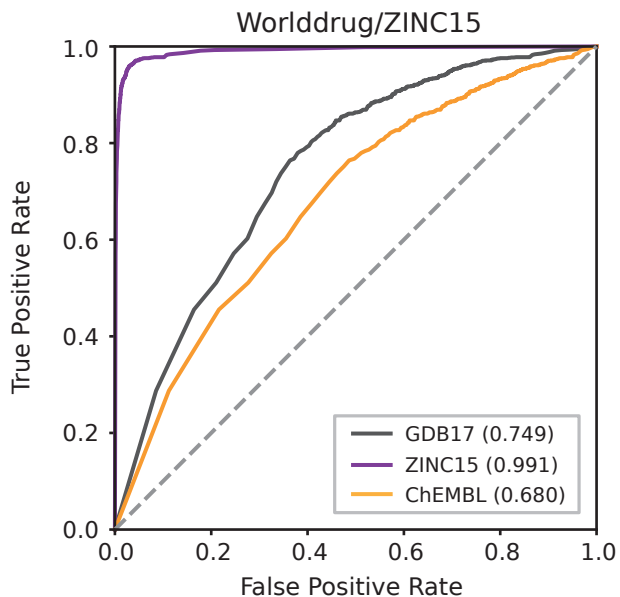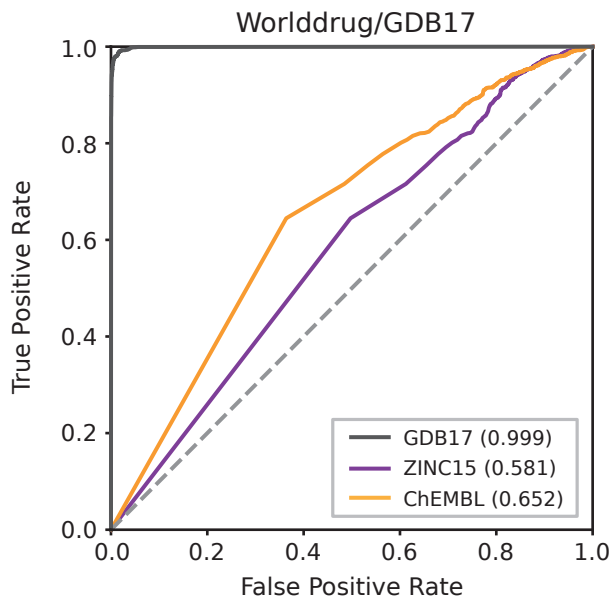

(c)

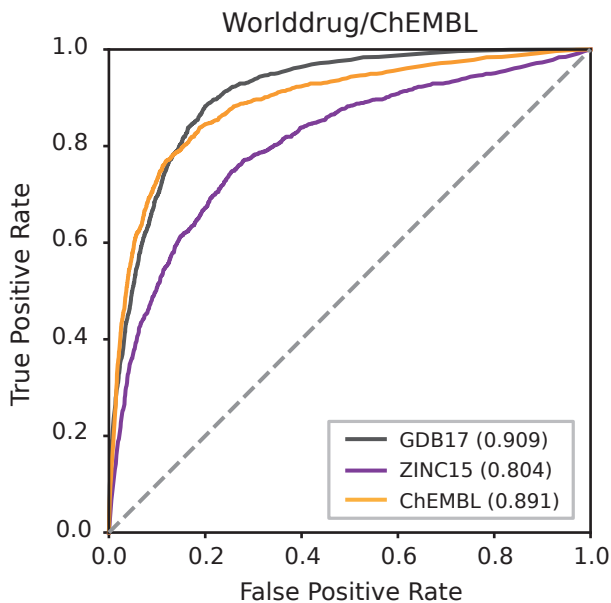

Supplement: SC-013-D1SC05248A-s002 [file SC-013-D1SC05248A-s002.pdf]
