# Supplementary material for: Drug-likeness scoring based on unsupervised learning
Source: Chem Sci. 2021 Dec 14;13(2):554–65. doi: 10.1039/d1sc05248a (PMC8729801; doi:10.1039/d1sc05248a)
Supplement: SC-013-D1SC05248A-s003 [file SC-013-D1SC05248A-s003.pdf]

Electronic Supplementary Material (ESI) for Chemical Science.
This journal is © The Royal Society of Chemistry 2021

Drug-likeness scoring based on unsupervised learning

Kyunghoon Lee‡[a], Jinho Jang‡[a], Seonghwan Seo‡[a], Jaechang Lim*[b], and Woo Youn Kim*[abc,]

‡*Equally contributed to the work.*

[a] *Department of Chemistry, KAIST, 291 Daehak-ro, Yuseong-gu, Daejeon 34141, Republic of Korea*

[b] *HITS incorporation, 124, Teheran-ro, Gangnam-gu, Seoul 06234, Republic of Korea.*

Email: jaechang@hits.ai

[c]*KI for Artificial Intelligence, KAIST, 291 Daehak-ro, Yuseong-gu, Daejeon 34141, Republic of Korea*

E-mail: wooyoun@kaist.ac.kr

Hyperparameters of the models

Table 1: Hyperparamter of the RNN and GCN Models

model	batch size	epoch	learning rate (lr)	lr decay	dropout rate
RNN	400	100	0.0001	0.99	0.2
GCN	100	determined by cross-validation	0.001	0.998	0.2

Effect of Transfer Learning

Here, we show the performance of the language model with and without transfer learning. The bold text indicates the highest AUROC values.

Table 2: The AUROC value of the three models including the RNN model with and without transfer learning and the GCN model as a baseline.

Model	FDA/GDB17	FDA/ZINC15	FDA/ChEMBL
RNN without transfer learning	0.824	0.795	0.767
RNN with transfer learning	**0.971**	0.923	**0.806**
GCN (supervised, baseline)	0.749	**0.991**	0.680

The transfer learning improved the performance of the RNN model for all test sets. However, the RNN model without the transfer learning still performed better than the GCN model for all cases except ZINC15. In the main text, we used the results from the RNN model with the transfer learning.

AUROC values of five randomly sampled negative sets

Table 3 we present the mean AUROC values of the GCN model along with the standard deviations for the five different negative training sets randomly sampled from the same data type. The standard deviations for each test were small compared to the mean values, indicating that an intra-domain dependency due to the use of a small fraction of the whole data set does not appear.

Table 3: The mean AUROC values from the five times evaluations of the GCN model trained with different training sets.

Model	FDA/GDB17	FDA/ZINC15	FDA/ChEMBL
GCN (TCC)	0.681 ± 0.061	0.991 ± 0.001	0.659 ± 0.015

PU-classification based TCC model

We applied a PU-learning method to examine whether the fixed negative set can lead to undesirable data bias in the model performance. The PU learning employed here uses a labeled positive set and an unlabeled set instead of the negative set. In this case, we set the ZINC15 as unlabeled set, since the molecules in ZINC15 can be either positive or negative in real experiments. The PU learning can selectively assign a reliable negative set from the unlabeled set according to the molecular features extracted from the learning process.

Specifically, we used the method proposed in the paper [49]: Fusilier *et al*, "Detecting positive and negative deceptive opinions using PU-learning." *Information processing & management*, 2015, **51**, 433–443. Here, it collects reliable negatives by removing false negatives from the unlabeled set using the probability output of the TCC model trained with the positive set and the unlabeled set. It assigns a data point as a reliable negative when the corresponding output probability is smaller than a threshold. The detail procedure works as follows:

1. Train the TCC model with the 2,833 Worlddrug molecules (positives) and the randomly sampled 10,000 ZINC molecules (unlabeled samples).
2. After training, get the output probability of the ZINC molecules. Remove the samples whose output probabilities are larger than a threshold value. Those removed molecules are considered as false negatives. The remaining molecules are considered as reliable negative. Here, we set the threshold value equal to 0.2.
3. Then, train the TCC model again with the same Worlddrug molecules and the remaining ZINC molecules.
4. Infer once again the output probability of each ZINC molecule assigned as the reliable negative. Repeat the procedure in step 2 to remove molecules whose output probabilities are higher than 0.2. The remaining molecules are considered as the new reliable negatives.
5. Repeat the step 3 and 4 until the number of the reliable negative set converges or becomes less than the number of the positive set.

Figure 1 summarizes the performance of the GCN-based TCC model trained with Worlddrug/ZINC15 with and without the PU learning described above. The result with the PU learning showed similar performance and data dependency with those of the original GCN model.

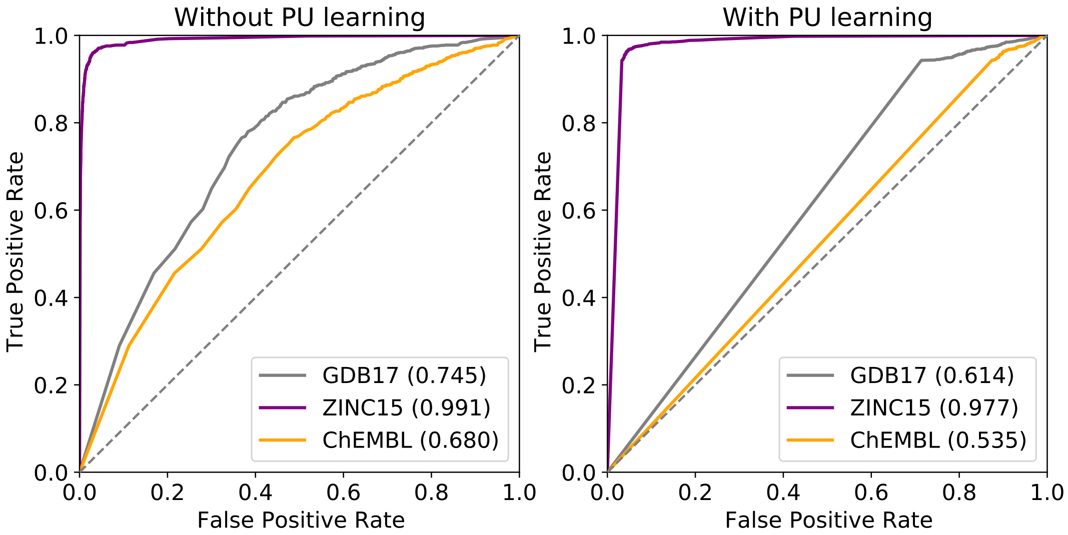

Figure 1: The ROC curves of the TCC models w/wo the PU learning on various test sets. The models were trained with Worlddrug/ZINC15. The values in the legend represent the AUROC values of each ROC curve. Both models still showed the highest performance on FDA/ZINC15, and their performances severely degraded on other test sets.

Negative set dependency of the GCN model performance

In the main article, we examined the data dependency of the deep learning models which were trained with the Worlddrug/ZINC15 dataset as positive and negative sets, respectively. In the case of the RNN model, it only requires the positive set. However, the GCN-based classification model needs both the positive and negative sets. Therefore, we further studied to examine if the strong data dependency of the GCN model still holds as we use different types of training sets. We prepared two more training sets for that purpose; Worlddrug/GDB17

and Worlddrug/ChEMBL. Similar to the preparation procedure of the Worlddrug/ZINC15 dataset, we used 2,833 molecules selected randomly from each of GDB17 and ChEMBL as the negative set. We noted that no molecule in the prepared training sets appears in the test sets. All the training data is available in Github https://github.com/SeonghwanSeo/DeepDL.

Figure 2 shows the ROC curves of the TCC models that are trained with different training sets. As we have expected in the introduction, the model showed the highest performance on the test set that was similar to the training set. For example, the model trained with Worlddrug/GDB17 showed the AUROC of 0.999 for FDA/GDB17. Likewise, the model trained with Worlddrug/ChEMBL showed the highest AUROC value of 0.891 for the FDA/ChEMBL test set. In contrast, the model performance significantly degraded on the other test sets, which are different from the training set. It should be noted that the ROC curves and the corresponding AUROC values significantly vary depending on the training set, showing the dependency of the classification model on the type of negative set.

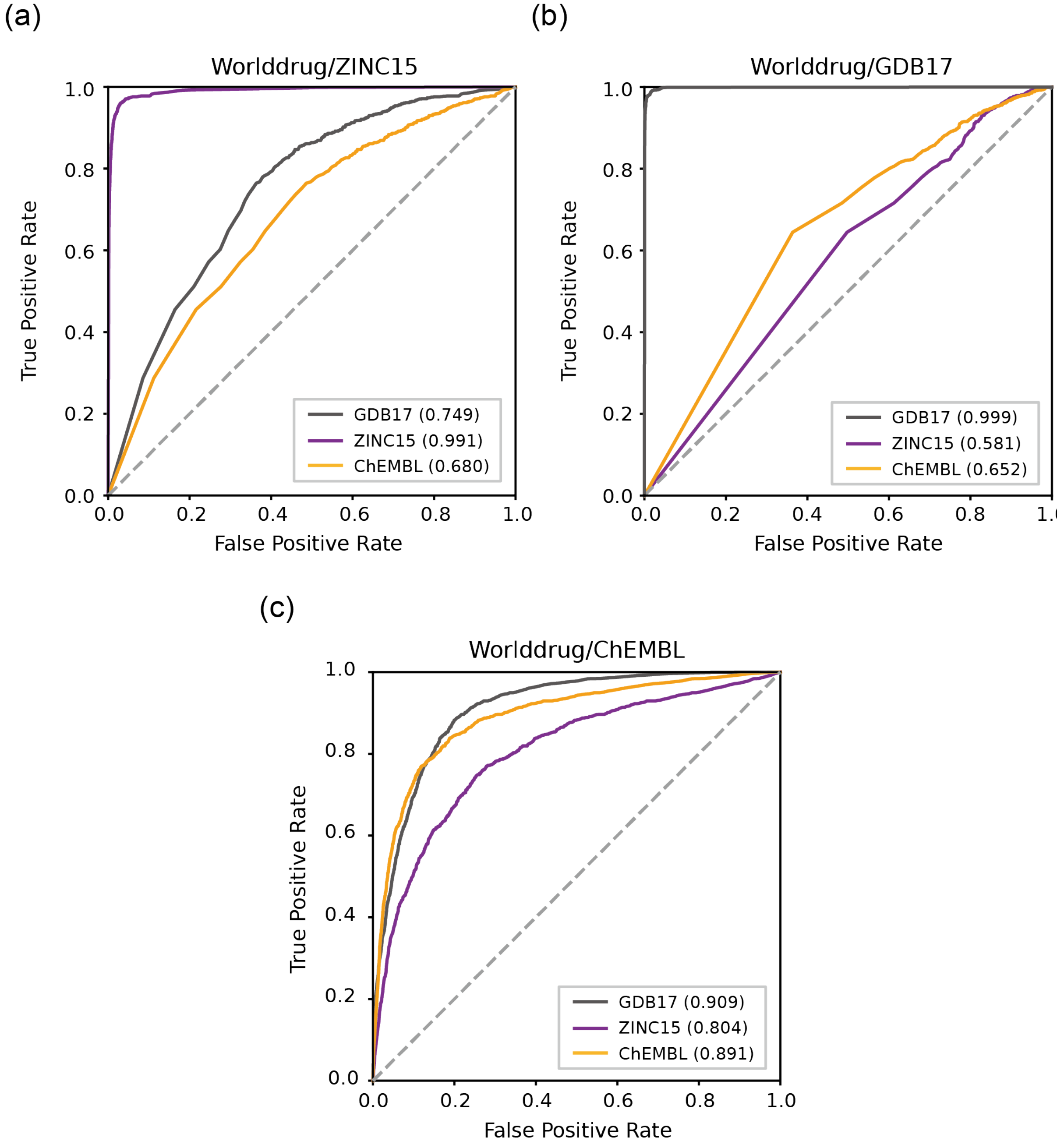

Figure 2: The ROC curves of the GCN-based TCC models on various test sets. The dotted line is the ROC curve of the random two-class classification. The numbers in the legend indicate the AUROC values of each ROC curve. (a) The ROC curves of the original GCN model shown in the main article, which was trained with Worlddrug/ZINC15. (b) The ROC curves of the GCN model trained with Worlddrug/GDB17. (c) The ROC curves of the GCN model trained with Worlddrug/ChEMBL.
